# Editorial: Integrative genetics and multi-omics of complex human disorders

**DOI:** 10.3389/fgene.2025.1574431

**Published:** 2025-02-21

**Authors:** Hongsheng Gui, Christopher J. Lessard, Jinhui Liu, Miaoxin Li, Indra Adrianto

**Affiliations:** ^1^ Center for Health Policy and Health Services Research, Henry Ford Health, Detroit, MI, United States; ^2^ Behavioral Health Services and Psychiatry Research, Henry Ford Health, Detroit, MI, United States; ^3^ Department of Psychiatry, College of Human Medicine, Michigan State University, East Lansing, MI, United States; ^4^ Henry Ford Health + Michigan State University Health Sciences, Detroit, MI, United States; ^5^ Genes and Human Disease Research Program, Oklahoma Medical Research Foundation (OMRF), Oklahoma City, OK, United States; ^6^ University of Oklahoma Health Sciences Center, Oklahoma City, OK, United States; ^7^ Department of Gynecology, The First Affiliated Hospital of Nanjing Medical University, Nanjing, Jiangsu, China; ^8^ Department of Genetics and Biomedical Informatics, Zhongshan School of Medicine, Sun Yat-sen University, Guangzhou, Guangdong, China; ^9^ Key Laboratory of Tropical Disease Control (Sun Yat-sen University), Ministry of Education, Guangzhou, Guangdong, China; ^10^ Center for Precision Medicine, Sun Yat-sen University, Guangzhou, Guangdong, China; ^11^ Department of Public Health Sciences, Center for Bioinformatics, Henry Ford Health, Detroit, MI, United States; ^12^ Department of Dermatology, Center for Cutaneous Biology and Immunology Research, Henry Ford Health, Detroit, MI, United States; ^13^ Immunology Research Program, Henry Ford Cancer Institute, Henry Ford Health, Detroit, MI, United States; ^14^ Department of Medicine, College of Human Medicine, Michigan State University, East Lansing, MI, United States

**Keywords:** genetics, genomics, transcriptomics, proteomics, multi-omics, bioinformatics, genome-wide association studies

Over the past two decades, genome-wide association studies (GWASs) have reported numerous susceptibility loci that provide useful insights into the molecular etiology of complex human disorders. However, elucidating the underlying genomic and molecular mechanisms of these disorders and how to apply these findings in clinical settings have been limited and challenging. Integration of genetics and multi-omics data (including but not limited to transcriptomics, proteomics, metabolomics, and microbiomics) offers a comprehensive understanding of the complexity of biological systems. The objective of this Research Topic is to provide cutting-edge research in the field of integrative genetics and multi-omics of complex disorders.

In this Research Topic, we selected multi-omics studies in type 2 diabetes mellitus (T2DM) (He et al.), psoriasis (Deng et al.), Alzheimer’s disease (AD) (Wang et al.), and chronic obstructive pulmonary disease (COPD) (Zou et al.). We also included a novel method for integrating multi-omics data called 3Mint (3 Multi-omics integrative tool) (Unlu Yazici et al.). Overall, these studies utilized multi-omics data to gain insights into the underlying molecular mechanisms of human disorders.

In the first article, He et al. utilized microarray and RNA-seq datasets from the Gene Expression Omnibus (GEO) database (GSE21321 and GSE15932) and performed differential gene expression and bioinformatics analyses to identify diagnostic genes associated with neutrophil extracellular traps (NETs) in T2DM. They observed five NETs-related diagnostic genes (*ITIH3*, *FGF1*, *NRCAM*, *AGER*, and *CACNA1C*) that have high diagnostic power for T2DM with the area under the curve (AUC) values of >0.7 for all genes. However, only two genes (*FGF1* and *AGER*) were validated in the blood of T2DM and control groups by qRT-PCR. The precise mechanisms of how these genes lead to T2DM are not fully understood and warrant further investigations.

The second study in our Research Topic utilized multiple bioinformatics methods to identify molecular lysosomal biomarkers for psoriasis. Deng et al. first identified potential lysosomal modulators via differential expression analysis between psoriasis and normal samples of bulk RNA-seq datasets from the GEO database (GSE13355 and GSE14905), then performed feature filtering via integrative machine learning model with GSE30999, GSE34248, GSE41662, and GSE53552 as validation datasets. They further validated the hub lysosomal genes at the bulk and single-cell levels by adding a single-cell RNA-seq dataset (GSE150672) and assessed the association of hub genes with the pathogenesis of psoriasis. Finally, they inferred the clinical significance of molecular subtypes based on hub lysosomal genes. They reported and validated *S100A7*, *SERPINB13*, and *PLBD1* as potential diagnostic biomarkers for psoriasis. These genes are likely involved in regulating cell communication between keratinocytes and fibroblasts via the PRSS3-F2R receptors, which provide potential therapeutic targets for psoriasis.

For the third article, Wang et al. integrated multiple-omics data to identify shared genetic architectures among cognition-related phenotypes and AD. They obtained GWAS summary statistics from the International Genomics of Alzheimer’s Project (IGAP), Social Science Genetic Association Consortium (SSGAC), Center for Neurogenomics and Cognitive Research (CNCR) CTGlab, and GWAS Catalog. For the expression quantitative trait locus (eQTL) analysis, they utilized resources from the CommonMind Consortium (CMC) and Genotype-Tissue Expression (GTEx). They first conducted a cross-trait linkage disequilibrium score (LDSC) regression to assess the genetic correlations of AD with the four cognition-related phenotypes (educational attainment, cognitive performance, childhood intelligence, and intelligence). They then performed local genetic correlation analysis using Heritability Estimation from Summary Statistics (HESS) to measure the similarity between a pair of traits driven by genetic variants and a pairwise analysis of GWAS (GWAS-PW) to evaluate the local correlations and associated SNPs of each LD-independent segment via a Bayesian colocalization framework. They also performed Mendelian randomization to determine causal relationships between the genetically predicted cognition-related phenotypes and AD and the eQTL analysis to evaluate whether the shared risk loci of AD and cognition-related phenotypes could regulate gene expression. After conducting transcriptome-wide association studies (TWAS), colocalization, and fine-mapping, they identified 11 pleiotropic risk loci of AD and cognition-related phenotypes and determined *TSPAN14*, *FAM180B*, and *GOLGA6L9* as the most credible causal genes of AD and cognition-related phenotypes. This study highlights the shared genetic basis of AD and cognition-related phenotypes, which can lead to new early prevention strategies for AD.

Next, Zou et al. assessed the causal association between COPD, major depressive disorder (MDD), and gastroesophageal reflux disease (GERD) using Mendelian randomization. They utilized GWAS summary statistics for these phenotypes from the FinnGen, United Kingdom Biobank, and Psychiatric Genomics Consortium (PGC) databases. They performed bidirectional Mendelian randomization and eQTL analyses and observed that MDD is likely to play a mediator role in the effect of GERD on COPD. Further functional mapping and annotation (FUMA) analysis revealed 15 genes associated with the progression of GERD-MDD-COPD. This study provides evidence that MDD plays a vital role in the GERD-COPD pathway, which highlights the importance of mental health assessment in patients with GERD and COPD.

Finally, Unlu Yazici et al. developed a novel integrative method for multi-omics data analysis by combining information from gene expression (mRNA), microRNA (miRNA), and methylation data named 3Mint. This method infers the statistical relationships among these omics data to create sets of pairs (miRNA-methylation/CpG features) and groups of highly correlated mRNAs with both miRNA and CpG pair, generate scoring for each group, and construct a machine-learning classification model based on the top-ranked groups. They also showed that 3Mint was able to classify breast cancer molecular subtypes with a smaller set of genes in comparison to the miRcorrNet tool (Yousef et al., 2021) while achieving similar performance metrics (95% accuracy). This method provides insights into how CpG methylation, miRNA, and targeted genes contribute to the disease of interest.

Overall, an integrative analysis of genetics and multi-omics aims to unravel the pathophysiology of complex disorders by evaluating the complex interacting components of biological systems. This can have a translational impact since a deeper mechanistic understanding will lead to better diagnosis, prevention, and intervention. While this field of study is promising, its implementation into clinical settings remains challenging and requires standardized data generation and Research Topic, robust methods to process and analyze the data, comprehensive validation via functional and clinical studies, training for clinicians to interpret and utilize multi-omics data in the practice, and ethical (data privacy and safety) considerations, and regulatory review and approval processes. Researchers need to address these aspects to pave the way for the integration of multi-omics into healthcare.
